# Mitochondrial dysfunction and metabolic reprogramming in acute kidney injury: mechanisms, therapeutic advances, and clinical challenges

**DOI:** 10.3389/fphys.2025.1623500

**Published:** 2025-08-06

**Authors:** Meiling Cao, Xueqi Zhao, Fang Xia, Mingyue Shi, Danyang Zhao, Lei Li, Hongkun Jiang

**Affiliations:** ^1^ Department of Neonatology, The First Hospital of China Medical University, Shenyang, Liaoning, China; ^2^ Department of Pediatrics, The First Hospital of China Medical University, Shenyang, Liaoning, China; ^3^ Department of Orthopaedic Surgery, Shengjing Hospital of China Medical University, Shenyang, Liaoning, China

**Keywords:** acute kidney injury (AKI), energy metabolic reprogramming, mitochondrial dysfunction, proximal tubular cells (PTC), therapeutic implications

## Abstract

Acute kidney injury (AKI) is a clinical syndrome associated with considerable morbidity and mortality. Despite therapeutic advancements, renal recovery and long-term outcomes remain suboptimal. Understanding the pathogenesis of AKI and identifying strategies to prevent its progression have become critical global health priorities. Mitochondrial dysfunction and changes in cellular energy metabolism play key roles in the pathophysiology of AKI. In patients with AKI, proximal tubular cells (PTCs) commonly exhibit impaired mitochondrial biogenesis, characterized by dysregulated mitochondrial dynamics, reduced fusion, and increased fission. Additionally, autophagy dysfunction may occur, contributing to compromised fatty acid β-oxidation (FAO) and subsequent energy deficits. To resolve this energy crisis, under the regulation of multiple signaling pathways, including AMP-activated protein kinase, mechanistic target of rapamycin complex 1, sirtuins, peroxisome proliferator-activated receptor alpha, peroxisome proliferator-activated receptor-γ coactivator 1α, and hypoxia-inducible factor-1 alpha, surviving PTCs may undergo a temporary shift toward glycolysis-dominant energy metabolism. This adaptive metabolic reprogramming is frequently associated with the activation of the pentose phosphate pathway and the suppression of gluconeogenesis. However, a sustained impairment of fatty acid oxidation (FAO) and continued reliance on glycolysis can result in the accumulation of lipids and glycolytic intermediates. This, in turn, may trigger inflammatory responses, promote epithelial-mesenchymal transition, impair tubular repair mechanisms, and contribute to the development of renal fibrosis. Collectively, these pathological processes facilitate the progression from acute kidney injury (AKI) to chronic kidney disease (CKD). Although interventions aimed at enhancing mitochondrial biogenesis, restoring mitochondrial and FAO homeostasis, and employing remote ischemic preconditioning have demonstrated potential in mitigating AKI progression, further investigation is required to address unresolved concerns related to their safety and clinical efficacy.

## 1 Introduction

Acute kidney injury (AKI) is a common and clinically significant syndrome characterized by an abrupt decline in renal function, typically indicated by elevated serum creatinine levels or reduced urine output (oliguria) (Hoste et al., 2018; Ronco et al., 2019). Epidemiological studies reported a high incidence of AKI across various populations. Despite the potential for renal function to return to baseline following appropriate treatment, long-term outcomes remain poor, with the condition contributing to considerable mortality and posing a substantial burden on global healthcare systems (Ronco et al., 2019; Gupta et al., 2024; Levey et al., 2020).

Mitochondria, as central organelles responsible for cellular energy production, play a key role in supporting diverse metabolic and physiological processes (Gewin, 2021). In the kidney, proximal tubular cells (PTCs) are particularly reliant on mitochondrial fatty acid β-oxidation (FAO) and oxidative phosphorylation (OXPHO) to meet their high energy demands (Han et al., 2016). These metabolic pathways generate significant amounts of adenosine triphosphate (ATP), which is essential for fueling the active transport and reabsorption functions of tubular epithelial cells (TECs) (Gewin, 2021; Houten et al., 2016).

In AKI, mitochondrial energy metabolic reprogramming plays a key role in disease pathogenesis. Mitochondrial dysfunction, characterized by impaired FAO and disrupted quality control mechanisms, has been frequently observed in affected patients (Freitas-Lima et al., 2020). Energy metabolic reprogramming in TECs is primarily driven by an imbalance in energy homeostasis, leading to a compensatory shift from FAO-dependent OXPHO to a glycolysis-dominant metabolic state. This adaptive shift resembles the Warburg effect commonly reported in tumor biology (Li Z. et al., 2021; Lan et al., 2016; Ma et al., 2022).

This metabolic transition is often accompanied by activation of the pentose phosphate pathway (PPP), which may contribute to cellular defense against oxidative stress induced by reactive oxygen species (ROS), as well as a concomitant suppression of gluconeogenesis (Scantlebery et al., 2021; van der Rijt et al., 2022). The enhanced glycolytic activity serves as a transient energy source, primarily sustaining the viability and limited functional capacity of the remaining TECs (van der Rijt et al., 2022). However, prolonged failure to reestablish normal mitochondrial metabolism may promote the progression of AKI to chronic kidney disease (CKD) (Shen et al., 2020).

Furthermore, mitochondrial dysfunction is closely implicated in AKI pathogenesis through the activation of inflammatory signaling cascades, which may lead to PTC apoptosis and necrosis (Jiang et al., 2020; Sun et al., 2019). These processes contribute to maladaptive repair, renal fibrosis, and ultimately the development of CKD.

Although advances in current treatment strategies have improved short-term outcomes, targeted therapies for AKI continue to face significant limitations. Changes in cellular metabolic pathways contribute to the progression of renal dysfunction. Early correction of energy metabolic reprogramming in TECs, along with the restoration of mitochondrial function, is considered a potentially valuable therapeutic approach to reduce the severity and long-term consequences of AKI (Ishimoto and Inagi, 2016).

This review outlines the metabolic transitions observed during AKI, emphasizing the underlying pathophysiological mechanisms and key regulatory pathways implicated in mitochondrial dysfunction and energy metabolism. In addition, current strategies for targeted therapeutic intervention are examined, along with their associated challenges and limitations.

## 2 AKI and mitochondrial energy metabolism

### 2.1 Basic structure and functions of mitochondria

Mitochondria are dynamic, double-membraned organelles typically exhibiting an ellipsoidal or rod-shaped morphology. Their structural components include the outer mitochondrial membrane (OMM), inner mitochondrial membrane (IMM), mitochondrial matrix, and intermembrane space (IMS) (Giacomello et al., 2020; Wang et al., 2024). The OMM serves as a selective barrier that regulates the diffusion of molecules and participates in mitochondrial signaling pathways. The IMM, consisting of the inner boundary membrane and cristae, is characterized by its inward folds that increase surface area for metabolic processes. This membrane provides the structural platform for OXPHO and the assembly of the, ETC (Trotta and Chipuk, 2017).

The mitochondrial matrix, enclosed by the IMM, supports several key metabolic processes, including the tricarboxylic acid (TCA) cycle and the later stages of glycolysis. The IMS plays a key role in numerous cellular functions, including protein and lipid biosynthesis, metal ion homeostasis, redox regulation, oxidative protein folding, initiation of apoptosis, and the maintenance of mitochondrial dynamics and structural integrity.

### 2.2 The key role of mitochondria in energy metabolism

The kidney exhibits a high resting metabolic rate, supported by a substantial mitochondrial content and elevated oxygen consumption. Different types of cells have different requirements for ATP, podocytes, endothelial cells and mesangial cells mainly use glucose for their energy supply, while renal tubular cells mainly use free fatty acids, glutamine, pyruvate, citrate and lactate as substrates for fuel supply through aerobic respiration (Scholz et al., 2021). Noteworthy primarily target the tubular epithelial cells especially the highly metabolically active proximal tubular segment (Isaka et al., 2011). PTCs are the most energy-demanding cells, which efficiently use FAO as a fuel source. This energy demand is primarily met through FAO and OXPHOS (Chung et al., 2019). FAO occurs predominantly within mitochondria and involves a sequence of cyclic reactions that convert fatty acids (FA) into acetyl-coenzyme A (acetyl-CoA). During this process, flavin adenine dinucleotide (FAD) is reduced to FADH_2_, and nicotinamide adenine dinucleotide (NAD^+^) is reduced to NADH (Foster, 2012). These reducing equivalents subsequently enter the mitochondrial electron transport chain, where their oxidation drives ATP production via OXPHOS. Species differences have been well documented for PPARα-regulated genes, such as those involved in peroxisome biogenesis and peroxisomal FAO (Lawrence et al., 2001; Sasaki et al., 2019).

### 2.3 Aberrant changes in mitochondrial structure and function during AKI

#### 2.3.1 Mitochondrial dysfunction

**FIGURE 1:**
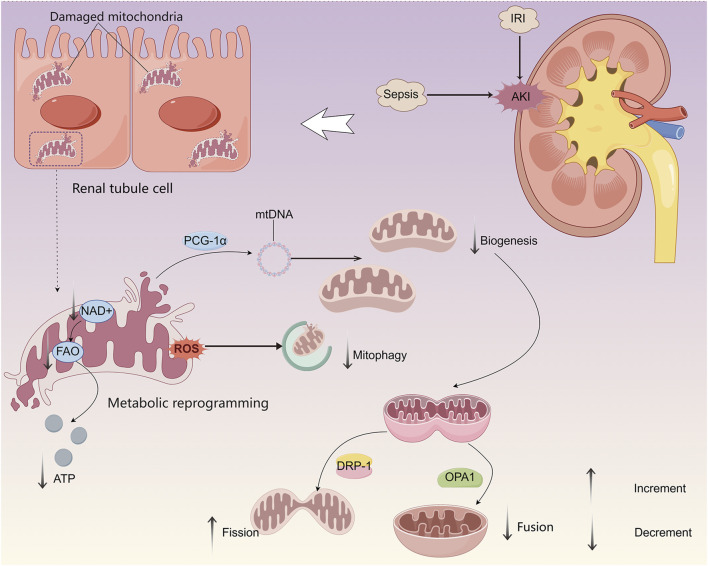
Mitochondrial dysfunction and its impact on energy metabolism in renal TECs during AKI. During AKI, mitochondrial dysfunction in TECs is characterized by impaired biogenesis, decreased mitochondrial fusion, increased fission, and dysregulated mitophagy, resulting in compromised mitochondrial quality control. These changes lead to a metabolic shift from FAO to glycolysis, serving as a transient compensatory mechanism to sustain minimal ATP production necessary for TEC survival.

Mitochondrial dysfunction in AKI is characterized by impairments in mitochondrial biogenesis, attenuation of mitophagy, disrupted mitochondrial dynamics, and reduced OXPHO activity (Figure 1) (Emma et al., 2016). Increased oxidative stress (OS) and excessive production of ROS are closely associated with the regulation of mitophagy (Zhu et al., 2023; Levine and Kroemer, 2019). This selective degradation of damaged mitochondria is mediated by key regulatory proteins, including PTEN-induced putative kinase 1, BCL2 interacting protein 3, and FUN14 domain-containing protein 1, and is essential for maintaining mitochondrial quality control (Quirós et al., 2016; Onishi et al., 2021; Lin et al., 2023). Upregulation of mitophagy represents an early cellular response aimed at promoting survival. However, excessive or sustained mitochondrial damage triggers pathological overactivation of mitophagy, contributing to cell death and tissue injury (Aggarwal et al., 2016).

During AKI, dysfunctional mitochondria can induce the expression of peroxisome proliferator-activated receptor-γ coactivator 1α (PGC-1α), which facilitates mitochondrial biogenesis and supports the maintenance of mitochondrial structure, function, and quantity. This adaptive response may contribute to reduced cell death and attenuation of lipid accumulation (Cheng et al., 2018). In sepsis-induced AKI models, proximal tubular cell-specific knockdown of PGC-1α has been associated with exacerbated renal injury and disease progression (Tran et al., 2011).

As highly dynamic organelles, mitochondria depend on continuous cycles of fission and fusion to preserve network integrity, regulate organelle quantity and quality, and adapt to fluctuating energy demands. Mitochondrial dynamics is very nuanced, and a balance between fission and fusion is critical depending on the cellular environment/state (Fan et al., 2024; Wai and Langer, 2016). Mitochondrial fission is promoted by the activation and translocation of dynamin-related protein 1, whereas mitochondrial fusion is mediated by optic atrophy 1, whose function may be disrupted by proteolytic cleavage, OPA1 is repressed by proteolysis, leading to dysregulation of mitochondrial fusion. Dysregulation of these dynamic processes can result in mitochondrial fragmentation, further contributing to cellular dysfunction during AKI (Qin et al., 2019).

#### 2.3.2 Dysfunction of mitochondrial energy metabolism

A reduction in the expression of peroxisome proliferator-activated receptor alpha (PPARα), a key regulator of FA homeostasis, has been associated with decreased activity of carnitine palmitoyltransferase 1 (CPT1), thereby impairing the transport of FAs into the mitochondrial matrix (Chung et al., 2018). This disruption may result in impaired oxidation of long-chain FAs and contribute to cellular necrosis, accompanied by downregulation of enzymes associated with FAO (Freitas-Lima et al., 2020; Erpicum et al., 2018).

Glycolysis may briefly enhance energy production and offer partial protective effects in the setting of AKI, but its prolonged activation has been linked to adverse renal outcomes. Although glycolysis can sustain basic cellular functions under stress conditions, persistent reliance on this pathway, along with the accumulation of lactate, promotes renal fibrosis (Hu et al., 2020). The combined effects of dysregulated FAO and sustained glycolytic activity contribute to the progression from AKI to CKD through multiple interrelated mechanisms (Li Z. et al., 2021). Enhanced glycolysis in a short period compensates for impaired energy production and exerts partial renal-protective effects. However, long-term shut down of FAO and enhanced glycolysis lead to inflammation, lipid accumulation, and fibrosis (Zhu et al., 2022).

#### 2.3.3 Enhancement of OS

ROS in AKI primarily originate from mitochondrial activity and nicotinamide adenine dinucleotide phosphate (NADPH) oxidase complexes (Cristofano et al., 2021; Zorov et al., 2014). Hypoxia and subsequent reperfusion serve as key initiating factors for ROS overproduction. During AKI, dysregulation of the antioxidant defense system impairs the clearance of excessive ROS, thereby disrupting redox homeostasis and promoting OS.

Elevated ROS levels can oxidatively modify mitochondrial lipids and proteins, resulting in functional disruption of the electron transport chain (ETC.) and increased permeability of the mitochondrial membrane. These changes ultimately compromise mitochondrial bioenergetics and contribute to cellular injury during the course of AKI (Hall et al., 2013).

### 2.4 The role of mitochondrial dysfunction in the pathogenesis of AKI

Mitochondrial dysfunction plays a key role in the pathophysiological progression of AKI. During AKI, mitochondria frequently exhibit morphological abnormalities, including swelling and fragmentation, which can facilitate the release of pro-apoptotic mediators and trigger the opening of the mitochondrial permeability transition pore. These processes lead to apoptosis and necrosis of PTCs, thereby aggravating renal injury and promoting AKI progression (Sun et al., 2020). Damaged mitochondria release cardiolipin and other bioactive molecules that activate Nucleotide oligomerization domain (NOD)-like receptors, contributing to elevated levels of pro-inflammatory cytokines and chemokines and result in sustained renal inflammation and injury (Szeto et al., 2017). Mitochondrial damage persists beyond the acute ischemic phase, maintaining chronic inflammasome activation and ultimately leading to progressive renal fibrosis.

Under renal injury and OS conditions, excessive ROS generation and the release of pro-apoptotic factors such as apoptosis-inducing factor impairs mitochondrial membrane potential and promotes both caspase-dependent and caspase-independent apoptotic pathways. There are two main caspase-dependent apoptotic pathways of apoptosis: the intrinsic pathway and the extrinsic pathway. The intrinsic pathway is triggered by disruptions in the cellular microenvironment, demarcated by mitochondrial outer membrance permeabilization, and precipitated mainly by caspases 3 (Sanz et al., 2023). The extrinsic pathway is initiated by the binding of proapoptotic ligands to death receptors on the cell surface, leading to the formation of the death-inducing signaling complex (DISC) and the activation of caspases (Qiao et al., 2024). Besides that, caspase-independent apoptotic pathway, known as the AIF/Endo G pathway, induces mitochondrial AIF release (Luo et al., 2021). Endo G acts as a modulator. Forced BNIP-3 expression by plasmid transfection results in mitochondrial Endo G release and nuclear translocation. BH3 domain of BNIP-3 interacted with anti-apoptotic protein to form dimers, which was able to promote the apoptosis (Luo et al., 2021; Liu et al., 2016).

Mitochondrial dysfunction disrupts communication between mitochondria and the endoplasmic reticulum (ER) (Igwebuike et al., 2020; Hasegawa and Inagi, 2020), contributing to tubulointerstitial inflammation and fibrosis. Various forms of renal injury are associated with ER stress and activation of the unfolded protein response (UPR), which can initially support cell survival by restoring ER and mitochondrial homeostasis through distinct signaling pathways (Zhou et al., 2025). However, when adaptive UPR mechanisms fail, they may instead promote cell death (Bartoszewska and Collawn, 2020). Due to hypoxia, mitochondrial dysfunction, and disordered nutrient-sensing pathways, the metabolic process in the TECs shifts from FAO to glycolysis. Long-term shut down of FAO and enhanced glycolysis lead to inflammation, lipid accumulation, and fibrosis. In AKI, TECs undergo autophagy-mediated phenotypic conversion to a pro-fibrotic state, leading to the secretion of fibroblast growth factor 2, which activates fibroblasts and contributes to maladaptive renal repair and fibrosis (Livingston et al., 2023).

Mitochondrial dysfunction not only contributes to the onset of AKI but also accelerates its progression. Renal injury is often accompanied by an intense inflammatory response (Kong et al., 2024). Necrotic tubular cells release damage-associated molecular patterns (DeWolf et al., 2022), which activate toll-like receptors on renal parenchymal and resident immune cells, stimulating the production of pro-inflammatory cytokines and chemokines. This cascade further promotes the transition from AKI to CKD. Although renal cells retain a capacity for self-repair, incomplete restoration of PTCs results in cell cycle arrest at the G2/M phase, a phenomenon that has been associated with tubulointerstitial fibrosis and irreversible renal damage. Such progression increases the risk of CKD and may ultimately lead to end-stage renal disease (Gewin, 2021; Zhao et al., 2021).

## 3 Mechanisms of mitochondrial energy metabolic reprogramming

### 3.1 Transition of energy metabolism pathways

Under physiological conditions, TECs actively absorb circulating FAs through specific transporter proteins, including CD36 and FA-binding proteins. Following entry into the cytoplasm, FAs are converted to acyl-coenzyme A (acyl-CoA) and subsequently processed by CPT1, a key rate-limiting enzyme located on the outer mitochondrial membrane. CPT1 catalyzes the formation of acyl-carnitines, which are transported into mitochondria where they are reconverted to acyl-CoA by CPT2 on the inner mitochondrial membrane. These acyl-CoAs then undergo β-oxidation to produce acetyl-CoA, which enters the TCA cycle for complete oxidation. The reducing equivalents NADH and FADH_2_ generated during FAO enter the, ETC, facilitating ATP production through OXPHO (Li et al., 2020). Very long-chain FAs require preliminary processing in peroxisomes before entering mitochondria for further oxidation (Houten et al., 2020).

Under normal metabolic conditions, the uptake, intracellular transport, and oxidation of FAs are tightly regulated to maintain lipid homeostasis, preventing lipid accumulation in the cytoplasm. This FAO-OXPHO-driven energy supply mode enables rapid ATP generation but requires high levels of oxygen, rendering TECs particularly susceptible to hypoxic injury. As a result, proximal tubules are more vulnerable to damage during episodes of AKI.

Along with FA metabolism, TECs serve as key sites of renal gluconeogenesis. Enzymes of renal gluconeogenesis, including phosphoenolpyruvate carboxykinase (PCK) and fructose-1,6-bisphosphatase (FBP), are predominantly expressed in TECs.

Under fasting or stress conditions, TECs utilize lactate as primary substrate for gluconeogenesis, which maintains systemic glucose homeostasis by coordinating with glucose reabsorption. Under normal physiological conditions, the glycolytic activity of TECs remains low, and ATP generated through aerobic glycolysis accounts for only a minor proportion (∼4%) of total ATP production (Guder and Ross, 1984).

During AKI, mitochondrial FAO and the transport of FAs into mitochondria are significantly impaired due to mitochondrial dysfunction and disruption of quality control mechanisms (Kang et al., 2015). In rat models of AKI induced by cisplatin, lipopolysaccharide (LPS), or ischemia-reperfusion (IR), significant reductions in ATP levels and intracellular lipid accumulation have been observed (Bhargava and Schnellmann, 2017). These models indicate reduced FA uptake in TECs, along with downregulation of key FAO enzymes such as CPT1 and long-chain acyl-CoA dehydrogenase (ACADL), as well as reduced expression and activity of PPARα, a central transcriptional regulator of FAO.

During both early and late reperfusion phases following IR injury, there is a notable increase in glucose uptake and glycolytic activity in the renal cortex. This metabolic shift is characterized by upregulation of rate-limiting glycolytic enzymes such as pyruvate kinase M2 isoform (PKM2) and hexokinase (HK), leading to elevated production of glycolytic end-products, including pyruvate and lactate (Li et al., 2020). Similar metabolic changes have been observed in isolated proximal tubules subjected to hypoxia/reoxygenation injury *ex vivo*, and increased activities of HK, PKM2, and lactate dehydrogenase (LDH) have been reported in mouse models of AKI (Li et al., 2020).

Collectively, these findings indicate a metabolic shift from FAO to glycolysis in TECs during AKI. Additionally, glucose is redirected into the PPP, which supports cellular repair and regeneration by producing NADPH and nucleotide precursors, and by maintaining intracellular levels of glutathione, a key antioxidant (Scantlebery et al., 2021). However, this shift is accompanied by a significant downregulation of gluconeogenic enzyme expression, resulting in hypoglycemia and lactate accumulation, which have been associated with increased severity of AKI and higher patient mortality (Legouis et al., 2020; Verissimo et al., 2022).

### 3.2 Modulation of key molecules

**FIGURE 2:**
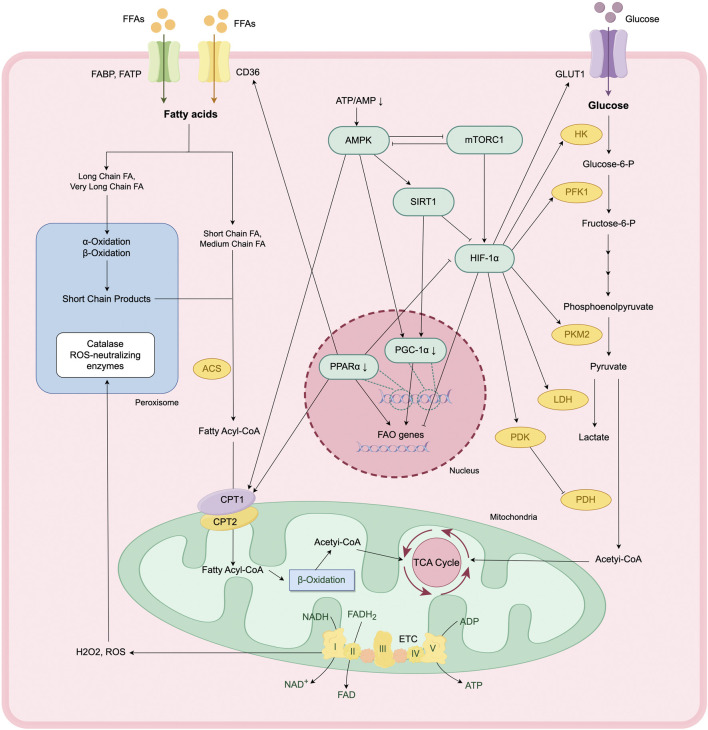
Metabolic reprogramming and regulation of key signaling pathways in AKI. In response to AKI, TECs undergo a metabolic transition from FAO- and OXPHO-dependent ATP production to a glycolysis-dominant state. This reprogramming is modulated by a complex signaling network, involving AMPK-SIRT1-PGC-1α axis, mTOR-HIF-1α pathway.

The metabolic reprogramming of TECs during AKI is regulated by an intricate signaling network involving AMP-activated protein kinase (AMPK), mechanistic target of rapamycin complex 1 (mTORC1), sirtuins (SIRTs), PPARα, PGC-1α, and hypoxia-inducible factor-1 alpha (HIF-1α). The activation of these pathways is initiated by different molecular signals depending on the etiology of AKI, as presented in Figure 2.

#### 3.2.1 HIF-1α

Renal hypoxia is a common feature across various AKI models, regardless of the initiating insult. The transcription factor HIF-1α plays a key role in mediating cellular responses to hypoxia and contributes to the metabolic reprogramming observed during AKI (Zhang et al., 2025). Under hypoxic conditions, the activity of cytoplasmic prolyl-hydroxylases is inhibited, preventing the hydroxylation and subsequent ubiquitination of HIF-1α, enhancing the stability of HIF-1α. Stabilized HIF-1α then translocates into the nucleus, where it forms a transcriptionally active heterodimer with HIF-1β. This complex binds to hypoxia response elements in the promoter regions of target genes, regulating the transcription of genes involved in metabolic adaptation and glycolysis (Lee S. H. et al., 2021).

HIF-1α inhibits the transcription of FA uptake genes (such as CD36) and β-oxidation rate-limiting enzyme genes (such as ACOX1, CPT1A), thereby suppress FAO activity in TECs (Li W. et al., 2021). While it enhances the transcription of glucose transport genes (such as GLUT1 and GLUT3) and key rate-limiting enzymes in glycolysis (such as PKM2 and HK2). Concurrently, HIF-1α upregulates the expression of LDH and pyruvate dehydrogenase kinase (PDK), promoting the conversion of pyruvate to lactate and inhibiting the entry of pyruvate into TCA cycle, further shifting energy metabolism toward glycolysis (Cai et al., 2020).

Along with its role in metabolic regulation, HIF-1α influences mitochondrial quality control. It mediates mitophagy, which provides cytoprotective effects by reducing apoptosis and ROS production during renal injury (Fu et al., 2020).

#### 3.2.2 PPARα/PGC-1α

PPARα is a ligand-activated nuclear transcription factor that is highly expressed in proximal renal tubules. It plays a key role in maintaining intracellular FA homeostasis by regulating the transcription of FAO-related enzymes in mitochondria and peroxisomes, and modulates cellular FA uptake through regulating the expression of CD36 and CPT1 (Emma et al., 2016). Additionally, PPARα exert indirect regulation over glycolysis by attenuating the transcriptional activity of HIF-1α (Iwaki et al., 2019). In murine models of ischemia-reperfusion injury (IRI) and drug-induced AKI, PPARα expression is significantly downregulated. Restoration of PPARα activity ameliorates metabolic dysregulation in these models (Lakhia et al., 2018). Conversely, reduced PPARα expression contributes to increased lipid accumulation and the development of fibrotic phenotypes in TECs, as demonstrated in CKD models.

PGC-1α is a key regulator of mitochondrial biogenesis (Fontecha-Barriuso et al., 2020). By activating nuclear receptors such as PPARα and ERRα, PGC-1α upregulates the expression of FAO key enzymes (e.g., CPT1A, ACOX1) and mitochondrial respiratory chain complex genes, maintaining energy homeostasis in TECs (Li and Susztak, 2018). Reduced expression of PGC-1α is consistently observed in animal models of AKI. TEC-specific knockout of PGC-1α results in exacerbated renal injury, whereas PGC-1α overexpression in TECs enhances NAD^+^ levels, thereby promoting mitochondrial biogenesis and mitophagy. These effects collectively reduce lipid accumulation and tubular injury and improve survival following renal IRI (Li et al., 2023; Tran et al., 2016). PGC-1α contributes to peroxisomal remodeling, further supporting its role in lipid metabolism and cellular homeostasis.

#### 3.2.3 mTOR/AMPK

The mechanistic target of rapamycin (mTOR) signaling pathway is a well-characterized nutrient-sensing pathway in the kidney. In response to external stimuli (e.g., hypoxia, ischemia, drug-induced injury), mTORC1 promotes the translation of HIF-1α, thereby driving a metabolic shift from oxidative phosphorylation to glycolysis (Cao et al., 2020; Cheng et al., 2014). Activation of mTOR pathway has been documented across multiple murine models of AKI. Pharmacological inhibition of mTORC1 suppresses excessive glycolysis and TEC proliferation, thereby ameliorating renal fibrosis (Cao et al., 2020; Cheng et al., 2014). In addition, mTORC1 inhibits mitophagy, further contributing to mitochondrial dysfunction (Wang et al., 2020).

AMPK, another key energy sensor in renal cells, negatively regulates mTOR signaling pathway. AMPK activation is triggered by a decrease in the ATP/AMP ratio, leading to SIRT1-mediated deacetylation and suppression of HIF-1α activity, thereby attenuating glycolysis (Clark and Parikh, 2021). AMPK enhances PGC-1α expression via SIRT1 activation, promoting mitochondrial biogenesis and mitophagy (Price et al., 2012). Furthermore, AMPK supports FAO by increasing CPT1 activity, which may contribute to improved ATP generation (Clark and Parikh, 2021).

Interestingly, the effects of AMPK activation appear to be time-dependent. Early-stage AMPK activation during AKI may fulfill elevated energy demands and support cellular repair mechanisms (Bao et al., 2019). However, during the later stages of injury, AMPK activity may be suppressed through mechanisms that remain to be fully elucidated, potentially exacerbating renal damage (Jin et al., 2020).

## 4 Specific role of mitochondrial energy metabolic reprogramming in AKI

**FIGURE 3:**
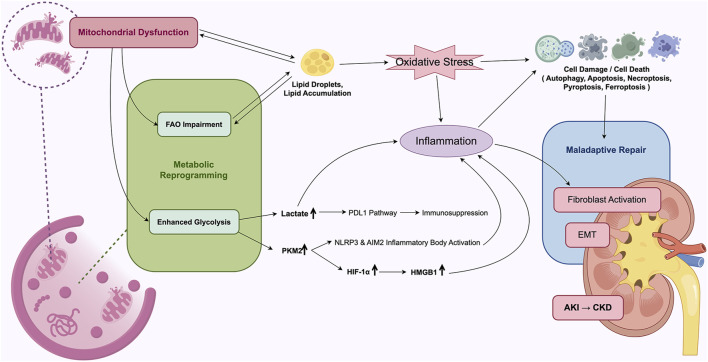
Mechanistic overview of mitochondrial energy metabolism reprogramming in AKI. Mitochondrial dysfunction and metabolic reprogramming leads to maladaptive repair of TECs, driving the development of AKI to CKD.

During AKI, metabolic reprogramming in TECs may temporarily compensate for deficits in energy supply, support cellular survival, and partially mitigate tissue damage, thereby facilitating early repair processes. However, sustained suppression of FAO along with persistent enhancement of glycolysis can result in intracellular lipid accumulation, activation of inflammatory pathways, and fibrogenesis, collectively contributing to the progression of AKI (Figure 3).

### 4.1 Exacerbation of renal TECs injury

Impairment of FAO results in the excessive accumulation of cytotoxic lipid species—such as triglycerides, free fatty acids (FFAs), cholesterol, and lysophosphatidylcholine—within TECs. This condition disrupts cellular homeostasis and contributes to cellular injury and death, a process commonly referred to as lipotoxicity (Jang et al., 2020; Todorović et al., 2021). Lipid accumulation adversely affects mitochondrial function, further impairing FAO, which reduces FFA utilization and promotes additional lipid deposition (Baek et al., 2022; Xu et al., 2022). This establishes a self-perpetuating cycle that may intensify renal injury. A similar bidirectional relationship is observed between mitochondrial dysfunction and lipid accumulation, whereby impaired mitochondria exhibit continuous electron leakage from complex I of the, ETC, resulting in the generation of ROS. These ROS trigger lipid peroxidation by stimulating the release of lipid radicals from membrane-associated triglycerides and phosphatidylcholine, thereby exacerbating lipid-induced cellular damage (Zhang et al., 2021).

The primary mechanism underlying renal injury associated with lipid overload appears to involve OS, but it is also closely linked to ER stress, defective autophagy, and inflammatory responses (Chen et al., 2018; Lee E. S. et al., 2021). Furthermore, metabolic reprogramming during AKI has been closely associated with multiple forms of programmed cell death in TECs, including apoptosis, necroptosis, pyroptosis, and ferroptosis.

Lactate accumulation may further amplify glycolytic activity by stabilizing HIF-1α, thereby creating a positive feedback loop that exacerbates mitochondrial dysfunction and cellular damage. Experimental studies have demonstrated that the glycolytic inhibitor 2-deoxyglucose can activate autophagy through the upregulation of SIRT3 and phosphorylated AMPK, reducing apoptosis in both sepsis-induced TEC injury models and LPS-stimulated HK-2 cells, ultimately improving cell viability (Tan et al., 2020; Tan et al., 2021). Additionally, pharmacologic agents such as shikonin (a PKM2 inhibitor) and dichloroacetate (a PDK1 inhibitor) have been shown to suppress TEC apoptosis and attenuate renal interstitial fibrosis (Wei et al., 2019).

### 4.2 Enhancing inflammatory response

Ischemic and toxic insults to the kidneys not only result in direct cellular injury but also elicit a robust inflammatory response. The dynamic balance between pro-inflammatory and anti-inflammatory pathways plays a critical role in determining the extent of subsequent renal damage and repair (Lee et al., 2024). TECs, while being primary targets of injury, also actively contribute to the regulation of the renal inflammatory microenvironment.

The glycolytic enzyme PKM2 exerts pro-inflammatory effects. Elevated PKM2 expression activates the transcription of HIF-1α and promotes the release of high mobility group box 1 (HMGB1) from activated macrophages, thereby amplifying sterile inflammation, particularly in sepsis-associated AKI, and enhancing TEC susceptibility to OS (Yang et al., 2014). In both IRI and sepsis-induced AKI models, HMGB1 release by TECs has been implicated in the promotion of renal inflammation (Zhao et al., 2023). Furthermore, PKM2 activation contributes to the intracellular accumulation of succinate, a signaling metabolite that stabilizes HIF-1α and promotes interleukin-1 beta (IL-1β) production during inflammatory responses (Tannahill et al., 2013). Inhibition of PKM2 within renal tissue has been associated with downregulation of HIF-1α, reduced TEC apoptosis, and amelioration of LPS-induced AKI (Yang et al., 2014). In sepsis-induced AKI models, PKM2 inhibition attenuates the activation of NLR family pyrin domain containing 3 (NLRP3) and absent in melanoma 2 (AIM2) inflammasomes, decreases the release of IL-1β, IL-18, and HMGB1 by macrophages, and ultimately improves survival outcomes (Xie et al., 2016).

Lactic acid, acting both as a metabolic intermediate and a signaling molecule modulates immune and inflammatory responses. In sepsis-induced AKI, the accumulation of lactic acid in renal tubules activates the programmed death-1/programmed death-ligand 1 (PD-1/PD-L1) axis, leading to lymphocyte apoptosis and contributing to immunosuppression (Xu et al., 2021). Conversely, other studies have demonstrated that lactic acid may partially restore PGC-1α activity, reduce the expression of pro-inflammatory cytokines including TNF-α, IL-1β, and IL-6, alleviate renal injury, and improve survival in murine models of sepsis-induced AKI (Takakura and Zandi-Nejad, 2019).

In addition, lipid-derived mediators produced from the autoxidation of intracellular polyunsaturated FAs, such as α-linolenic acid and docosahexaenoic acid (DHA), exhibit anti-inflammatory effects (Lin et al., 2020). Exogenous DHA supplementation has been shown to significantly modulate oxylipin profiles in ischemia-induced AKI, thereby enhancing renal tubular function. However, excessive intake of unsaturated FAs may increase the production of pro-inflammatory lipid mediators and potentially worsen renal injury (Zhang et al., 2025; Sonnweber et al., 2018).

### 4.3 Facilitation of renal fibrosis

Renal fibrosis represents the final common pathological outcome in most CKD cases, with epithelial injury and aberrant repair processes serving as central events in its progression. In AKI, TECs may undergo cytoskeletal reorganization and extracellular matrix (ECM) accumulation, transitioning from an energy-demanding epithelial phenotype to a less metabolically active interstitial phenotype—a process known as epithelial-mesenchymal transition (EMT) (Grande et al., 2015). In addition, a subset of injured TECs may become arrested in the G2/M phase of the cell cycle and adopt a pro-fibrotic phenotype (Yang et al., 2010). Beyond the impact of endothelial damage, peritubular capillary rarefaction, pericyte detachment, nephron loss, interstitial hypoxia, and persistent inflammation, metabolic disturbances in TECs—particularly defective FAO and lipid accumulation—have been identified as key contributors to EMT and the progression of renal fibrosis through maladaptive repair mechanisms (Miguel et al., 2021).

In fibrotic kidneys, both the expression of FAO-associated enzymes and the transcriptional regulator PPARα are significantly downregulated. This reduction correlates positively with the extent of lipid accumulation. Restoration of FAO in experimental models of renal fibrosis have significantly reduced interstitial fibrotic changes (Kang et al., 2015). Evidence from folic acid-induced nephropathy in mice indicates that impaired FAO, rather than intracellular lipid droplet accumulation, serves as the principal determinant of tubulointerstitial fibrosis (Lan et al., 2016). Moreover, in a transgenic mouse model with kidney-specific overexpression of CD36, increased triglyceride and long-chain FA accumulation was insufficient to induce renal fibrosis, further supporting the notion that reduced FAO activity—rather than lipid storage alone—is more directly associated with fibrogenic progression in TECs (Kang et al., 2015).

Enhanced glycolytic activity has been implicated in the activation of fibroblasts, whereas pharmacological inhibition of glycolysis has been shown to significantly attenuate renal fibrosis (Ding et al., 2017). In a folic acid-induced AKI model, interstitial fibroblasts were activated and proliferated by metabolizing lactic acid produced by TECs during the later stages of injury. Early intervention using glycolysis inhibitors to limit tubular lactate production effectively suppressed fibroblast activation and mitigated renal fibrosis (Shen et al., 2020). Furthermore, pro-inflammatory microenvironments can promote fibroblast activation and ECM deposition. In a unilateral ureteral obstruction-induced model of renal interstitial fibrosis, upregulated glycolysis in TECs and elevated lactate levels contributed to a hypoxic and acidic microenvironment that inhibited podocyte proliferation and differentiation, thereby accelerating fibrotic remodeling (Li et al., 2018).

### 4.4 Effects on renal hemodynamics and systemic metabolism

The interaction between metabolic shifts and renal hemodynamics/tubuloglomerular feedback (TGF) is one of the core mechanisms of AKI, which involves imbalance of energy metabolism, accumulation of metabolites, oxidative stress, inflammation and regulation of signal pathways (Wada et al., 2024; Fogo and Harris, 2025). Glomerular filtration rate (GFR) affects the tubular reabsorption load, and TECs maintain ion transport by consuming ATP, which accounts for 80% of renal oxygen consumption. Energy status of TECs (e.g., ATP/AMP ratio) reversely regulates glomerular blood flow via the AMPK pathway. Lactate produced by TEC glycolysis dilates the afferent glomerular arteriole, while long-chain acyl-CoA released from impaired FAO enhances vasoconstriction.

Metabolic alterations during AKI also exert profound impacts on systemic metabolism. Impaired FAO and enhanced glycolysis in TECs lead to increased production of lactate and pyruvate, which serve as substrates for hepatic gluconeogenesis. In I/R models, lactate released by the kidney enters the liver through portal vein, where it activates pyruvate carboxylase (PC) and phosphoenolpyruvate carboxykinase (PEPCK), thereby promoting glucose synthesis (Yu et al., 2021). Concomitantly, reduced renal clearance of insulin and glucagon in AKI results in an elevated glucagon/insulin ratio, which triggers hepatic cAMP/PKA pathway and promotes gluconeogenesis (He et al., 2025).

## 5 Intervention and treatment of mitochondrial energy metabolic reprogramming in AKI

### 5.1 Mitochondrial-targeted peptide D-Arg-dimethylTyr-Lys-Phe-NH2 (SS-31)

The mitochondrial-targeted peptide SS-31 has demonstrated significant protective effects in renal tubular cells by attenuating OS, thereby reducing apoptosis and necrosis (Zuo et al., 2020; Cho et al., 2010; Lin et al., 2019). This agent contributes to the preservation of tubular function and provides protection against ischemia-induced renal injury. SS-31 stabilizes mitochondrial structure and function, prevents the collapse of mitochondrial membrane potential, and inhibits calcium-induced opening of the mitochondrial permeability transition (MPT) pore. These effects collectively promote ATP synthesis and reduce tubular cell loss (Table 1).

**TABLE 1:** Mitochondria-targeted therapeutics.

Therapeutics	Mechanisms of action	Experimental model	Clinical condition	Limination
SS-31	Binds to cardiolipin, prevents peroxidase activity and improves mitochondrial respiration and ATP production; Inhibits cytochrome c release;	IRI-AKI, UUO, and DKD	Mitochondrial myopathy; Age-related skeletal muscle mitochondrial dysfunction	It is unknown if later administration of SS-31 can stop the progression of CKD.
MitoQ	ROS scavenger	IRI-AKI; Ins2(+/)−(AkitaJ) mice; Phase Ⅱclinical trials	Parkinson’s disease; Fatty acid disease; Hepatitis C; CKD	MitoQ induces rapid swelling and depolarization of mitochondria in PTCs

Resveratrol	Sirt-1 activator; Restores mitochondrial respiratory capacity and decreases the production of mtROS and lipid peroxidation	Hemorrhagic shock induces AKI and db/db mice	_	RSV has low bioavailability and solubility. The security of resveratrol application in human is not clear.
CPT1	Promotes FAO; maintain mitochondrial homeostasis	IRI-AKI	−	The shift from FAO to glycolysis reduces dependence on CPT1.
AICAR	Regulates the AMPK pathway	IRI-AKI and UUO	Heart Failure; T−cell leukemia/lymphoma	The impact of long-term use is not clear.
Rapamycin	Inhibits mTOR signal pathway, induces autophagy and reduces the release of inflammatory factors.	IRI-AKI; CI-AKI; SA-AKI	Cancer; Autoimmune Disease; Neurodegenerative Disease	Rapamycin has uncertain efficacy and potential adverse effects.
rIPC	Decreased kidney ROS production, preserved mitochondrial dynamics and mitophagy	IRI-AKI	Kidney transplantation; Heart surgery	Although AKI mice models revealed that rIPC effectively improved kidney dysfunction, the renoprotection effect of early or late-phase rIPC remains to be verified.

ATP, adenosine triphosphate; IRI-AKI, ischemic reperfusion induced acute kidney injury; UUO:unilateral ureter obstruction; DKD, diabetic kidney diseases; ROS, reactive oxygen species; Mdivi-1, Mitochondrial division inhibitor-1; Drp1, dynamin related protein 1; AMPK, AMP-activated protein kinase; mtROS, mitochondria reactive oxidative species; CKD, chronic kidney disease; MitoQ, mitochondrial coenzyme Q; CI-AKI, contrast-induced acute kidney injury; SA-AKI, sepsis associated acute kidney injury.

Moreover, SS-31 mitigates mitochondrial dysfunction and prevents subsequent cell death and inflammatory responses, which are implicated in the progression from AKI to CKD. By targeting upstream mitochondrial injury, SS-31 may serve as a therapeutic candidate for reducing long-term renal damage following AKI. It is not known if later administration of SS-31 can stop the progression of CKD (Szeto et al., 2017).

### 5.2 Mitochondria-targeted antioxidants

The targeted mitochondrial antioxidant mitoquinone (MitoQ) has demonstrated protective effects in AKI by attenuating OS and apoptosis. In models of renal IRI, pre-ischemic administration of MitoQ significantly reduces oxidative damage and the severity of renal injury (Dare et al., 2015). In db/db mice and angiotensin II-infused models, intraperitoneal injection of MitoQ alleviated excessive mitochondrial fission and restored mitochondrial autophagy, thereby mitigating tubular apoptosis and renal damage (Xiao et al., 2017; Zhu et al., 2021). Noteworthy recent *in vitro* data indicating that MitoQ causes mitochondrial swelling and depolarization in kidney tissue, but the doses were supraphysiological (Gottwald et al., 2018).

Resveratrol (RSV), a naturally occurring polyphenolic antioxidant, has indicated potential in improving mitochondrial function. RSV administration has been associated with increased mRNA and protein expression levels of PGC-1α and SIRT1, promoting mitochondrial homeostasis, restoring respiratory capacity, and attenuating cellular injury (Zhang et al., 2020). Unfortunately, this compound exhibits low bioavailability and solubility (Vesely et al., 2021). Clinical investigations of RSV in human populations remain in early stages, and further studies are needed to determine its efficacy and safety in patients with AKI.

### 5.3 FAO activators can improve mitochondrial energy metabolism in AKI

Impaired mitochondrial FAO in AKI represents a potential therapeutic target for mitochondrial-directed studies. Current strategies aimed at restoring FAO primarily involve compounds that activate the PPARα, PGC-1α, and AMPK signaling pathways (Console et al., 2020). Following renal IRI, FAO activity is significantly reduced. Modulation of CPT1 activity through pharmacological inhibition has been reported to act as a direct ligand for PPARα, thereby enhancing FAO levels and ameliorating fibrosis following IRI (Tanaka et al., 2011). Targeted modulation of CPT1 promotes PPARα-mediated FAO, protects TECs, and reduces renal injury in AKI models. Furthermore, restoration of energy homeostasis via PGC-1α activation in TECs attenuates both AKD and the progression to CKD.

Dysregulation of the mTOR and AMPK signaling pathways has been implicated in the metabolic reprogramming of TECs during AKI. Therapeutic modulation of this axis represents a promising approach to mitigating renal injury. Activation of AMPK using 5-aminoimidazole-4-carboxamide ribonucleotide (AICAR) enhances mitochondrial biogenesis, promotes mitochondrial FAO, and reduces renal damage in AKI models (Jin et al., 2020; McWilliam et al., 2021). In addition, both AICAR and RSV—a SIRT1 activator—have demonstrated efficacy in promoting mitochondrial adaptation and biogenesis, thereby reducing TEC injury and renal fibrosis in preclinical studies (Hong et al., 2017; Morigi et al., 2015; Xu et al., 2016). The impact of long-term use is not clear. Rapamycin, an mTOR inhibitor induces mitophagy and modulates cellular metabolism and proliferation. But rapamycin has uncertain efficacy and potential adverse effects (Moghadamnia et al., 2024).

Although FAO activators exhibit considerable therapeutic potential for AKI, the precise mechanisms by which FAO suppression promotes renal fibrogenesis remain incompletely understood. Moreover, the clinical translation of pharmacological agents targeting mitochondrial function and energy metabolism remains limited, highlighting the need for further investigation in both preclinical and clinical settings.

### 5.4 The potential value of remote ischemic preconditioning (rIPC) in preventing AKI in renal tissue within a clinical practice

The rIPC exerts organ-protective effects through two distinct temporal phases: an acute phase and a delayed phase (Jia et al., 2024; Bolli, 2000; Bolli, 2007). The rIPC is induced by transient episodes of ischemia and reperfusion in distant tissues, which can subsequently confer protection against future ischemic injury in target organs, including the kidneys (Cho et al., 2010; Jang et al., 2012; Jang et al., 2008). The acute phase of rIPC is primarily mediated by rapid post-translational modifications of pre-existing proteins and the activation of signal transduction cascades (Pei and Zhou, 2017). In contrast, the delayed phase involves transcriptional upregulation and synthesis of cytoprotective proteins, such as superoxide dismutase, heat shock proteins, and inducible nitric oxide synthase, along with modulation of anti-apoptotic pathways. Direct and indirect effects on mitochondria by IPC can result in the activation of AMPK, a master regulator of cellular metabolism. Changes in the activity of the posttranslational modifiers, SIRT1 and SIRT5, also contribute to the overall adaptive processes in cellular metabolism and mitochondrial functioning (Jackson et al., 2018). RIPC maintains mitochondrial quality through mitochondrial autophagy to clear dysfunctional mitochondria (Livingston et al., 2019), which is essential for restoring organ integrity. Wei et al. found that rIPC may attenuate mitochondrial dysfunction in AKI by inhibiting NOX4-ROS signaling (Long et al., 2022), rIPC protected kidney function and pathological injury and mitigated NADPH oxidase 4 (NOX4) upregulation in different AKI models (cisplatin, LPS and IRI). Livingston, M.J., et al. found that the mitophagy regulator PINK1 was activated during IPC. Knockdown of *PINK1* suppressed mitophagy and reduced the cytoprotective effect of IPC (Livingston et al., 2019). IPC also suppressed mitochondrial depolarization, improved ATP production, and inhibited the generation of reactive oxygen species.

The rIPC is regarded as a simple, non-invasive, cost-effective, and generally safe intervention with substantial potential for clinical application. Its acute phase has demonstrated efficacy in providing renal protection in various experimental models (Long et al., 2022). Although AKI mice models revealed that rIPC effectively improved kidney dysfunction, the renoprotection effect of early or late-phase rIPC remains to be verified (Wei et al., 2025). Besides that, the clinical evidence supporting delayed rIPC remains limited due to small sample sizes, and further large-scale studies are needed to evaluate its safety and therapeutic effectiveness.

**FIGURE 4:**
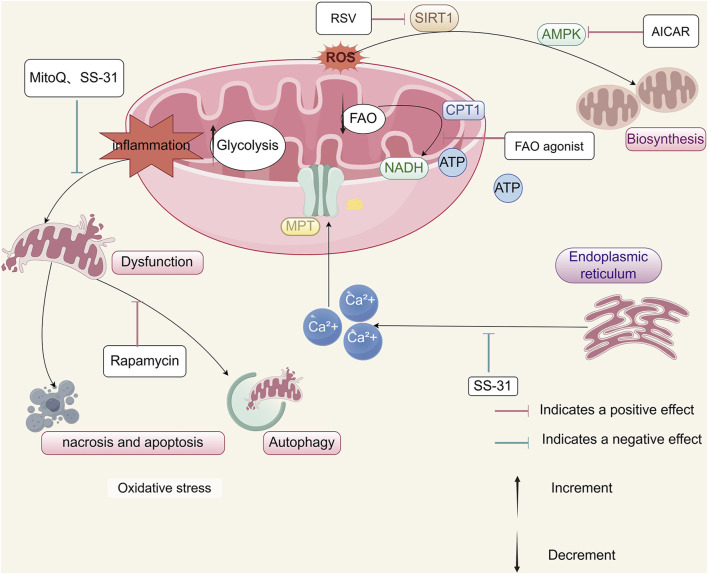
Therapeutic strategies targeting mitochondrial energy metabolism in AKI. Potential therapeutic approaches to mitigate mitochondrial dysfunction in AKI include: reduction of ROS using mitochondrial-targeted antioxidants such as SS-31 and MitoQ; inhibition of calcium-induced MPT pore opening by SS-31 to preserve ATP synthesis; enhancement of mitochondrial autophagy via rapamycin to maintain organelle quality; stimulation of mitochondrial biogenesis through RSV or AICAR; restoration of energy metabolism through FAO activation; and renal protection through rIPC induced by bilateral renal ischemia.

Among the molecular mediators implicated in rIPC, heat shock protein 70 (HSP70) possesses cytoprotective properties, particularly through inhibition of apoptosis (Stankiewicz et al., 2005). Experimental studies have indicated that rIPC upregulates intracellular HSP70 expression, which is subsequently released into the extracellular space and systemic circulation during stress conditions. HSP70 contributes to the protective mechanisms associated with ischemic preconditioning. Therefore, further research is warranted to clarify the role of HSP70 in rIPC-induced renal protection, including its regulatory mechanisms and potential therapeutic implications (Figure 4).

## 6 Conclusions and future prospects

The kidney, as the second most metabolically active organ in the human body, possesses a high mitochondrial density. These mitochondria serve not only as central sites for cellular energy metabolism but also as critical regulators of cell death pathways. Mitochondrial dysfunction and energy metabolic reprogramming are closely implicated in the onset and progression of AKI. This review has summarized current evidence regarding structural and functional changes in mitochondria, shifts in cellular energy metabolism, and recent advancements in the understanding of molecular mechanisms underlying these changes in AKI. Additionally, potential therapeutic strategies targeting mitochondrial dysfunction have been outlined, with emphasis on their relevance to AKI prevention and treatment.

Following AKI, TECs exhibit significant mitochondrial abnormalities, including disrupted dynamics of fission and fusion, impaired biogenesis, and altered morphology. These changes, regulated by a network of molecular pathways, lead to metabolic reprogramming characterized by downregulated FAO and enhanced glycolysis. This is accompanied by disturbances in membrane lipid and triglyceride metabolism, activation of the PPP, and suppression of gluconeogenesis. Although the upregulation of glycolysis offers transient metabolic compensation and oxidative stress resistance, sustained impairment of FAO results in lipid accumulation and progressive TEC injury, thereby promoting inflammation and fibrosis, ultimately resulting in poor renal outcomes. Early therapeutic interventions aimed at restoring mitochondrial function and enhancing FAO may prove to be more effective in halting disease progression rather than targeting downstream pathologies alone. Preclinical studies have indicated promising results using mitochondrial-targeted peptides, antioxidants, FAO activators, and rIPC in mitigating renal injury.

Despite the therapeutic potential of these strategies, several limitations and challenges remain. First, current knowledge of mitochondrial biology in the setting of AKI remains incomplete. The role of mitochondrial communication with other organelles, such as ER and lysosomes, requires further elucidation. Studies have shown that mitochondria-associated ER membranes (MAMs), the ER part directly connected to the mitochondria, participate in the pathogenesis of AKI by regulating calcium signaling, lipid metabolism and energy homeostasis (Igwebuike et al., 2020; Inagi, 2020). Second, existing research on metabolic reprogramming in AKI has predominantly focused on proximal tubular epithelial cells, while the metabolic responses of other renal cell types—including podocytes, endothelial cells, and interstitial cells—remain inadequately studied, to solve which single-cell metabolomics might be a promising method. Third, the precise mechanisms by which upstream and downstream regulatory molecules influence mitochondrial function and metabolic pathways are not fully understood. Circadian regulators such as brain and muscle arnt-like protein 1 (BMAL1) (Ye et al., 2022; Yang et al., 2024) may also interact with metabolic pathways in AKI. Additionally, the molecular basis through which FAO impairment contributes to renal fibrogenesis warrants further investigation.

Furthermore, although various mitochondrial-targeted interventions have demonstrated efficacy in preclinical models, their clinical translation remains limited. Challenges include inter-individual variability in metabolic responses due to heterogeneous AKI etiologies, dynamic shifts in metabolic states across AKI stages, and variability in inflammatory environments. Potential off-target effects and unforeseen adverse reactions also constrain the advancement of candidate therapeutics into clinical application. Identification of injury-specific therapeutic targets and optimization of intervention timing represent important areas for future research.

Despite these limitations, ongoing investigation is expected to yield deeper insights into the mitochondrial pathophysiology of AKI and support the development of more effective and clinically translatable therapeutic strategies.

## Abbreviations

AKI, Acute kidney injury; UPR, Unfolded Protein Response; ER, Endoplasmic reticulum; CKD, Chronic kidney disease; ESRD, End-stage renal disease; PTCs, proximal tubule cells; OXPHOS, oxidative phosphorylation; TECs, Tubular epithelial cells; FAO, Fatty acid β-oxidation; ATP, adenosine triphosphate; FAs, Fatty acids; PPARα, Peroxisome proliferator-activated receptor alpha; IR, Ischemia-reperfusion; CPT, Carnitine palmitoyltransferase; ACADL, Long-chain acyl-CoA dehydrogenase; HK, Hexokinase; PKM2, M2 isoform of pyruvate kinase; LDH, Lactate dehydrogenase; LPS, Lipopolysaccharides; PPP, Pentose phosphate pathway; GSH, Glutathione; 2-DG, 2-deoxyglucose; NAD+/NADH, Nicotinamide adenine dinucleotide; EMT, Epithelial-mesenchymal transition; HIF-1α, Hypoxia inducible factor-1; PHD, Prolyl-hydroxylases; VHL, Von-Hippel-Lindau; HRE, Hypoxia response elements; FADH2, Flavin adenine dinucleotide; PDK, Pyruvate dehydrogenase kinase; PGC-1α, Peroxisome proliferator-activated receptor-γ coactivator 1α; AMPK, AMP-activated protein kinase; AICAR, 5-Aminoimidazole-4-carboxamide ribonucleotide; PINK1, PTEN induced putative kinase 1; BNIP3, BCL2 interacting protein 3; FUNDC1, FUN14 domain-containing protein 1; HMGB1, High mobility group box 1; NLRP3, NLR family pyrin domain containing 3; AIM2, Absent in melanoma 2; TGF-β, Transforming growth factor-beta; SS-31, D-Arg-dimethylTyr-Lys-Phe-NH2; MitoQ, Mitoquinone; TNF-α, Tumor necrosis factor-α; OMM, mitochondrial membrane; IMM, inner mitochondrial membrane; IMS, mitochondrial intermembrane space; ETC, electron transport chain; ROS, reactive oxygen species; SIRT1, sirtuin1; FGF2, fibroblast growth factor 2; Glut1, Glucose transporter 1; FABP, fatty acid-binding protein; FBP, fructose-1,6-bisphosphatase; PCK, Phosphoenolpyruvate Carboxykinase; OS, oxidative stress; PD-L1, programmed cell death ligand 1; HSP70, the 70-kDa heat shock proteins; RSV, resveratrol; BRI, bilateral renal ischemia; DAMPs, damage associated molecular patterns; ADP, adenosine diphosphate; IL, interleukin; mtDNA, mitochondrial DNA; rIPC, remote ischemic preconditioning; PTEN, phosphatase and tensin homologue; FFA, free fatty acid; ECM, extracellular matrix; NOX4, Nicotinamide adenine dinucleotide phosphate oxidase 4; TGF, tubuloglomerular feedback; GFR, glomerular filtration rate; PC, pyruvate carboxylase; PEPCK, phosphoenolpyruvate carboxykinas.
